# A Synchrosqueezed Transform Method Based on Fast Kurtogram and Demodulation and Piecewise Aggregate Approximation for Bearing Fault Diagnosis

**DOI:** 10.3390/s24082502

**Published:** 2024-04-13

**Authors:** Yanlu Chen, Lei Hu, Niaoqing Hu, Jiyu Zeng

**Affiliations:** 1 College of Railway Transportation, Hunan University of Technology, Zhuzhou 412007, China; m21081101015@stu.hut.edu.cn (Y.C.);; 2 Hunan Provincial Key Laboratory of Health Maintenance for Mechanical Equipment, Hunan University of Science and Technology, Xiangtan 411201, China; 3 Laboratory of Science and Technology on Integrated Logistics Support, National University of Defense Technology, Changsha 410073, China

**Keywords:** fault diagnosis, time–frequency analysis, fast kurtogram, piecewise aggregate approximation, synchrosqueezed transform

## Abstract

Synchrosqueezed transform (SST) is a time–frequency analysis method that can improve energy aggregation and reconstruct signals, which has been applied in the fields of medical treatment, fault diagnosis, and seismic wave processing. However, when dealing with time-varying signals, SST suffers from poor time–frequency resolution and is unable to deal with long signals. In order to accurately extract the characteristic frequency of variable speed rolling bearing faults, this paper proposes a synchrosqueezed transform method based on fast kurtogram and demodulation and piecewise aggregate approximation (PAA). The method firstly filters and demodulates the original signal using fast kurtogram and Hilbert transform to reduce the influence of background noise and improve the time–frequency resolution. Then, it compresses the signal by using piecewise aggregate approximation, so that the SST can deal with long signals and, thus, extract the fault characteristic frequency. The experimental data verification results indicate that the method can effectively identify the fault characteristic frequency of variable-speed rolling bearings.

## 1. Introduction

Rolling bearing is one of the key components in rotating machinery but also one of the causes of mechanical failure. Therefore, rolling bearing fault diagnosis is of great significance. In practical engineering, rotating machinery is often in variable speed conditions; where the speed changes with time, and the frequency of bearings failure also vary with time. The fault signal is a kind of non-stationary signal. The time–frequency analysis method is an effective tool to analyze the non-smooth signal. Examples include linear time–frequency transformation methods such as Short-Time Fourier Transform (STFT) [1] and Continuous Wavelet Transform (CWT) [2], as well as nonlinear time–frequency analysis methods like the Wigner–Ville Distribution (WVD) [3] and Empirical Mode Decomposition (EMD) and its derivatives. However, these methods have various drawbacks. STFT and CWT suffer from the Heisenberg uncertainty principle, which means they cannot simultaneously achieve the best resolution in both time and frequency, leading to resolution discrepancies and energy divergence issues. While WVD improves the resolution of time–frequency representation, it generates cross-terms when dealing with multi-component signals, making it difficult to accurately extract signal features [4]. EMD suffers from endpoint effects and mode mixing problems. The classic time–frequency analysis does not work well for detecting time-varying fault characteristic frequency harmonics of the bearing due to the limitation of time–frequency resolution and noise interference [5].

In 2011, Daubechies et al. [6] proposed a method with the advantages of time–frequency reassignment (RM), namely synchrosqueezed transform (SST). SST rearranges the wavelet coefficients obtained from the Continuous Wavelet Transform, sharpening the time–frequency representation, thereby enhancing energy concentration and preserving the signal reconstruction characteristics. However, many studies have shown that SST’s performance in handling time-varying signals is unsatisfactory, with issues of energy divergence [7,8]. In response to these problems, many experts and scholars have further researched and applied SST to various fields. T. Oberlin et al. [9] introduced the Short-Time-Fourier-Transform-based Synchrosqueezing Transform (FSST), and later proposed the second-order SST based on STFT (FSST2). On this basis, Pham et al. [10] proposed the High-Order Synchrosqueezing Transform (HSST), which has been applied in the analysis of multi-component signals and achieves favorable results. Zhang [11] applied this method to the resonance band feature extraction of sound signals in planetary gearboxes. Li et al. [12,13] proposed the Adaptive SST (ASST) and the Adaptive SST based on STFT (AFSST), then extending them to the second order. It has been verified that this method not only improves the time–frequency concentrate on and resolution of multi-component signals but also separates their components. Yu et al. [14,15] successively proposed the Synchroextracting Transform (SET) and the Multisynchrosqueezing Transform (MSST), continuously improving time–frequency concentration. Furthermore, based on the Time-Reassigned Synchrosqueezing Transform (TSST) proposed by He et al. [16], the Time-Reassigned Multisynchrosqueezing Transform (TMSST) [17] was introduced by Yu, achieving good results in the analysis of fault frequencies in impact-type vibration signals. Xu et al. [18] improved the instantaneous frequency extraction of nonlinear, non-stationary, multi-component signals through Synchrosqueezing Short-time Fourier Transform. Wan et al. [19] proposed the Variational Mode Decomposition -SET (VMD-SET) and successfully applied it to the order tracking of a wind turbine gearbox without speedometer under time-varying conditions. Zhao et al. [20] proposed termed Frequency-Chirprate Synchrosqueezing-based Scaling Chirplet Transform (FCSSCT) and successfully applied it to a wind turbine planetary gearbox and bearing.

However, the above improved SST methods are based on the assumption of slow time variation or weak frequency variation in the signals. When the rolling bearings are operating at higher speeds, with significant background noise, the processing effect is still not satisfactory. Additionally, due to the large memory requirements and low computational efficiency of the Synchrosqueezing Transform method in signal processing analysis, it is not suitable for processing long signals. Taking this computer as an example, with a memory capacity of 64 GB, during the SST computation, there is a continuous need for instantaneous frequency estimation and calculation of time–frequency results. Based on multiple experimental tests, the maximum data size that SST can handle is 96,000. If the sampling frequency is 25,600 Hz and the signal duration is 3.75 s. For the extraction of fault characteristics in variable-speed rolling bearings, the information contained in a signal of this duration is not enough. However, longer signals provide more reliable information [21], especially when the rolling bearing signals are of long duration under variable-speed conditions, direct processing of short signals is not conducive to observing the changes in fault frequencies of rolling bearings over time.

To obtain reliable fault frequency diagnosis results for variable-speed rolling bearings, this paper proposes a bearing fault diagnosis method, synchrosqueezed transform based on fast kurtogram and demodulation and piecewise aggregate approximation. By using the fast spectral kurtosis method to identify the optimal frequency band and the Hilbert transform, the original signal is filtered and demodulated. This process not only filters and denoises the original signal but also enhances time–frequency concentration. Furthermore, piecewise aggregate approximation is applied to the filtered envelope signal. Compared to directly compressing the original signal, compressing the envelope signal reduces information loss. Finally, the compressed signal is processed using the Synchrosqueezing Transform method to extract fault characteristic frequencies. This method effectively reduces the influence of background noise and improves time–frequency representation. Furthermore, it addresses the issue of computational memory constraints, enabling Synchrosqueezing Transform to handle large volumes of vibration signals. Through comparative analysis of simulated signals and experimental signal processing results, the proposed method demonstrates its effectiveness in identifying fault frequencies of rolling bearings under variable-speed conditions. Compared to STFT, classical SST, and MSST, it exhibits the best time–frequency resolution.

## 2. Basic Principles of Synchrosqueezed Transform

Before introducing the method proposed in this paper, let us first review the SST based on STFT.

In general, the STFT of the signal can be represented as follows:

(1) $$G(t,\omega )=\int_{-\infty }^{\infty }g(u-t)x(u)e^{-i\omega u}du,$$

where g() is the window function. Then, the STFT with an additional phase offset is given by

(2) $$G(t,\omega )=\int_{-\infty }^{\infty }g(u-t)x(u)e^{-i\omega (u-t)}du.$$

Assuming a time-varying signal model,

(3) $$s(t)=A(t)e^{i\varphi (t)},$$

where A(t) represents the instantaneous amplitude, $\varphi \left(t\right)$ represents the instantaneous phase, and its first derivative $\varphi^{\prime }\left(t\right)$ represents the instantaneous frequency. If the signal is weakly time-varying, i.e., there exists a sufficiently small ε such that for any time t, $\left|A^{\prime }(t)\right|\leq \varepsilon$ and $\left|\varphi^{\prime \prime }(t)\right|\leq \varepsilon$, according to the Taylor expansion, we can obtain the instantaneous amplitude and phase at time t:

(4) $$\begin{array}{l} A(u)=A(t)\\ \varphi (u)=\varphi (t)+\varphi^{\prime }(t)(u-t). \end{array}$$

If higher-order terms in the Taylor expansion $O(A^{\prime }(t))$ and $O(\varphi^{\prime \prime }(t))$ are neglected, the signal can be represented as:

(5) $$s(t)=A(t)e^{i(\varphi (t)+\varphi^{\prime }(t)(u-t))}.$$

Therefore, the STFT result of s(t) can be represented as follows:

(6) $$\begin{array}{ll} G(t,\omega ) & =\int_{-\infty }^{+\infty }g(u-t)A(t)e^{i\left(\varphi (t)+\varphi^{\prime }(t)(u-t)\right)}e^{-i\omega (u-t)}du\\ & =A(t)e^{i\varphi (t)}\int_{-\infty }^{+\infty }g(u-t)e^{i\left(\varphi^{\prime }(t)(u-t)\right)-i\omega (u-t)}du\\ & =A(t)e^{i\varphi (t)}\hat{g}\left(\omega -\varphi^{\prime }(t)\right), \end{array}$$

where $\hat{g}()$ represents the Fourier transform of the window function and $\mathrm{supp}(\hat{g})\in \left[-\Delta_{\omega },\Delta_{\omega }\right]$. From the above equation, it can be observed that the energy distribution of the STFT result is centered around the instantaneous frequency $\omega \in \left[\varphi^{\prime }(t)-\Delta ,\varphi^{\prime }(t)+\Delta \right]$. According to the theory of SST, for any (t,ω) and G(t,ω)≠0, the estimated instantaneous frequency of the STFT of s(t) is as follows:

(7) ω^(t,ω)=Re∂tG(t,ω)iG(t,ω)=Re∂tA(t)eiφ(t)g^ω−φ′(t)iA(t)eiφ(t)g^ω−φ′(t)=ReA(t)eiφ(t)g^ω−φ′(t)iφ′(t)iA(t)eiφ(t)g^ω−φ′(t)=φ′(t),

where Re() represents the real part. Then, the result of the SST of s(t) is:

(8) $$Ts(t,\eta )=\int_{-\infty }^{+\infty }G(t,\omega )\delta (\eta -\hat{\omega }(t,\omega ))d\omega .$$

Substituting Equations (6) and (7), we can obtain:

(9) $$\begin{array}{ll} Ts(t,\eta ) & =\int_{-\infty }^{+\infty }A(t)e^{i\varphi (t)}\hat{g}\left(\omega -\varphi^{\prime }(t)\right)\delta \left(\eta -\varphi^{\prime }(t)\right)d\omega \\ & =A(t)e^{i\varphi (t)}\delta \left(\eta -\varphi^{\prime }(t)\right)\int_{-\infty }^{+\infty }\hat{g}\left(\omega -\varphi^{\prime }(t)\right)d\omega \\ & =2\pi g(0)A(t)e^{i\varphi (t)}\delta \left(\eta -\varphi^{\prime }(t)\right). \end{array}$$

Comparing the results of STFT and SST, it can be observed that, through the operation of SST, the blurred energy of the STFT result can be concentrated within a region near each modal instantaneous frequency trajectory, thereby improving the time–frequency concentration.

The following expression demonstrates that SST can reconstruct the original signal:

(10) $$\begin{array}{ll} \int_{-\infty }^{+\infty }Ts(t,\eta )d\eta & =\int_{-\infty }^{+\infty }\int_{-\infty }^{+\infty }G(t,\omega )\delta \left(\eta -\hat{\omega }(t,\omega )\right)d\omega d\eta \\ & =\int_{-\infty }^{+\infty }G(t,\omega )\int_{-\infty }^{+\infty }\delta \left(\eta -\hat{\omega }(t,\omega )\right)d\eta d\omega \\ & =\int_{-\infty }^{+\infty }G(t,\omega )d\omega \\ & =(2\pi g(0))s(t). \end{array}$$

Thus,

(11) $$s(t)={\left(2\pi g(0)\right)}^{-1}\int_{-\infty }^{+\infty }Ts(t,\omega )d\omega .$$

## 3. The Proposed Method

**Figure 1:**
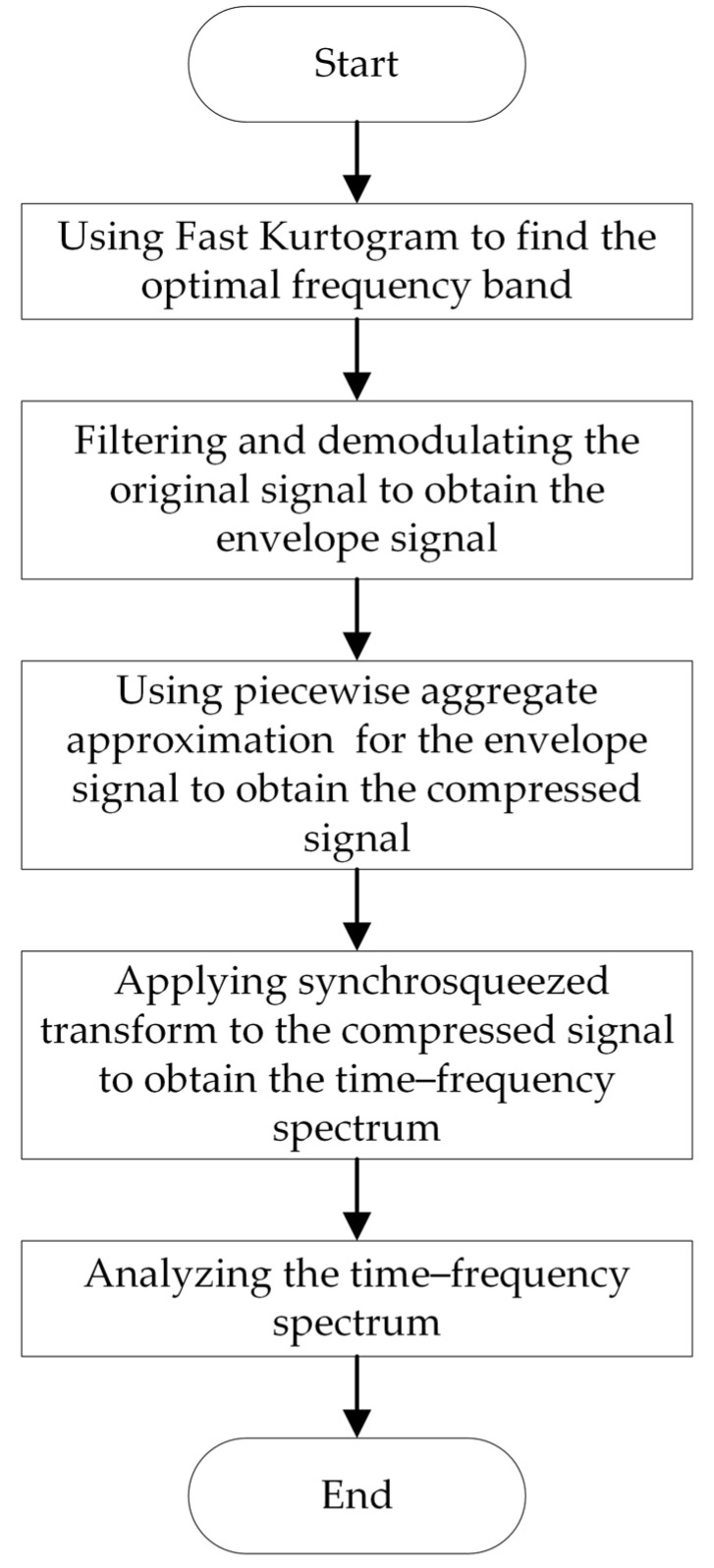
The method flowchart.

In summary, compared to other time–frequency analysis methods, SST has the advantages of improving time–frequency concentration and signal reconstruction. However, SST requires performing a Short-Time Fourier Transform in the calculation process. According to Equation (6), the length of the STFT window G(t,ω) is determined by the length of the signal *s*. The size of the signal data to be processed depends on the computer’s computational memory, thus limiting SST to handling only short signals [11,12,13,14]. However, in practical engineering applications, vibration signals from variable-speed rolling bearings are mostly long signals. Therefore, the challenge lies in how to use the SST concept to extract time-varying fault characteristic frequencies from long-term signals. Additionally, SST may perform poorly in extracting fault frequencies from signals with strong frequency variations. To address these issues, this paper proposes a bearing fault diagnosis method—synchrosqueezed transform based on fast kurtogram and demodulation and piecewise aggregate approximation. The processing flow of this method, as shown in Figure 1, includes five steps: optimal frequency band selection, filtering and demodulation, piecewise aggregate approximation, SST, and time–frequency spectrum analysis.

### 3.1. Optimal Frequency Band Selection

Rolling bearing vibration signals are modulated signals. Selecting the optimal frequency band can improve the efficiency and accuracy of signal analysis, making it essential. The fast kurtogram is an effective signal analysis tool first proposed by Antoni [22] in 2007, and its effectiveness has been demonstrated in subsequent applications. The fast kurtogram can reduce the interference of background noise on the signal and identify the high-frequency and low-frequency components of the signal. It first utilizes a tree-structured filter bank to filter the vibration signal and obtain the distribution of kurtosis, and then it identifies the optimal filtering frequency bands from it. By selecting the optimal frequency bands, it creates conditions for enhancing the modulation signals of low fault frequencies and high natural frequencies in the subsequent steps.

### 3.2. Filtering and Envelope Demodulation

Filtering is conducted based on Section 3.1, followed by envelope demodulation of the filtered signal using the Hilbert transform method. The analytical signal of the vibration signal can be represented by taking the original vibration signal as the real part and its Hilbert transform as the imaginary part. Therefore, the analytical signal of the filtered vibration signal is as follows:

(12) y=s+iH(s),

where *s* is the filtered signal, H(s) is the Hilbert transform of *s*, and, thus, the envelope of the signal is $\left|y\right|$. From the above, it can be deduced that the frequency after envelope transformation only contains positive frequency components.

### 3.3. Piecewise Aggregate Approximation

Piecewise aggregate approximation (PAA) is a signal compression method [23]. PAA can compress large amounts of time series data while preserving the original characteristics of the data as much as possible. Assuming the test signal is $x=\left\{x_{i}\right\}$, the sampling frequency is *f*_s_ and the signal length is *L*; PAA first defines a constant *w*, then divides the sample sequence *x* into *N* segments, and finally calculates the arithmetic mean of each segment sequence.

(13) $$p_{n}=\frac{1}{w}\sum_{i=w(n-1)+1}^{nw}x_{i}\;n\in \left[1,L\right].$$

Thus, the compressed new sequence can be obtained as $p=(p_{1},p_{2},\cdot \cdot \cdot ,p_{N})$. *w* is also known as the size of the PAA window. The larger the *w*, the smaller the amount of compressed data but also the more information lost.

For rolling bearing vibration signals, the size of the PAA window *w* can be selected based on the characteristic fault frequencies of the bearing. The equivalent sampling frequency of the compressed signal *p* is *f*_s_/*w*. Assuming the maximum value of the characteristic fault frequency of the bearing is *f*_max_ and also assuming that *K* times the fault characteristic frequency is retained in the signal, then, according to the sampling theorem, the equivalent sampling frequency of the compressed signal must satisfy:

(14) $$f_{S}/w\geq 2.56K\cdot f_{\max}.$$

That is, the PAA window size needs to satisfy

(15) $$w\leq f_{S}/\left(2.56K\cdot f_{\max}\right).$$

According to the above, PAA is not only a simple algorithm but also can keep as many original features of the data as possible while compressing a large amount of time series data, which can address the low computational efficiency of SST.

### 3.4. Synchrosqueezing Transform

According to the principle of synchrosqueezed transform, as illustrated in Section 1, the compressed signal in Section 3.3 is processed. This involves applying the SST algorithm to the compressed signal to obtain its time–frequency representation and then plotting a spectrogram to illustrate the characteristics of the signal in both time and frequency domains.

### 3.5. Time–Frequency Spectrum Analysis

Compare the processed results with the predicted results and analyze the effectiveness of the processing results.

## 4. Simulation Signal Validation

In this section, we validate the effectiveness of the proposed method using a simulated signal of variable speed rolling bearing outer race fault. Assume the rolling bearing rotation frequency is $f_{r}(t)$ and the inner race speed is $\omega =2\pi f_{r}(t)$, where the angle rotated can be considered as the integral of the speed over time. Thus, throughout the entire rotation process, the accumulated angle of the inner race can be represented as $\theta_{i}=\int_{t_{1}}^{t_{n}}\omega \left(t\right)dt$. According to the bearing motion mechanism, we can obtain the accumulated rotation angles of the cage and rolling elements relative to the center of the axis:

$$\begin{array}{l} \mathrm{Cage}:\theta_{c}=\frac{D-d}{2D}\cdot \theta_{i}\\ \mathrm{Rolling}\;\mathrm{element}:\theta_{b}=-\frac{D-d}{2d}\cdot \theta_{i} \end{array}$$

where *D* is outside diameter and *d* is the bore diameter.

**Figure 2:**
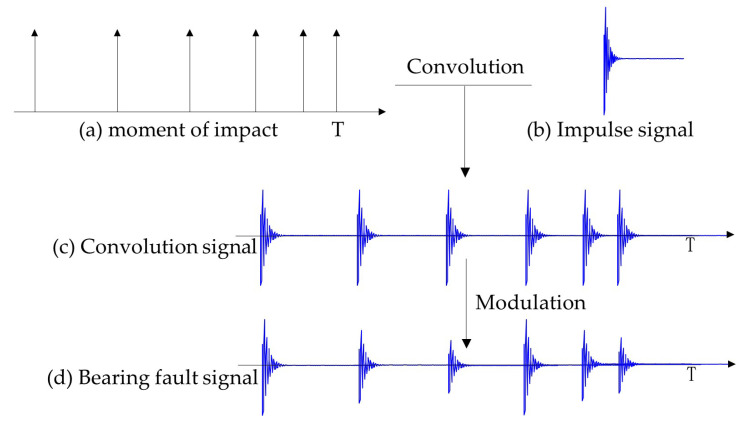
Schematic diagram of bearing simulation principle.

In theory, the vibration signal of a rolling bearing can be considered as a repetitive impulse signal composed of a series of exponentially decaying pulses. Therefore, firstly, in the time domain, convolution of a unit impulse signal with an attenuated signal is performed at each occurrence of the impulse. Then, modulation is applied based on different fault conditions. Finally, the vibration signal corresponding to the respective fault is obtained through convolution and modulation, as shown in Figure 2. Here, the exponentially decaying signal is defined as $s=e^{-Bt}\cos(2\pi f_{n}t)$, where B is the decay exponent and $f_{n}$ is the natural frequency.

We can calculate the moments of vibration impact based on the rotated angle. Taking a rolling element as an example: when the outer race has a fault, every time the cage completes one revolution the rolling element impacts the fault point on the outer race once. Therefore, based on the relationship between $\theta_{c}$ and 2π, we can calculate the timing of each impact. Assuming there are Z rolling elements, let $\theta_{c}$ be the angle rotated by the first rolling element with the cage. Then the angle rotated by the n-th rolling elements with the cage is $\theta_{n}=\theta_{c}+2\pi \left(n-1\right)/Z$. When the cage completes one revolution, each rolling element impacts the fault point on the outer race once. We can determine the timing of each impact based on the relationship between the rotation angle of each rolling element with the cage $\theta_{m}$ and 2π.

Given the rotation angle of each component during operation, calculating the moments of impact is as follows: Firstly, we take the modulus $\mathrm{mod}(\theta_{m},2\pi )$ of the rotation angle $\theta_{m}$ with respect to 2π. If the component completes an integer number of revolutions, the modulus is 0; otherwise, it is non-zero. Within every two revolutions, the modulus gradually increases, reaching its maximum when the rotation angle is closest to the next revolution and its minimum just after completing a full revolution. Hence, by taking the difference in the modulus values, abrupt changes occur when passing through the complete revolution position. This allows us to determine the moments of impact.

Using the established bearing fault model described above, simulations were conducted for the rolling bearing with the parameters, as shown in Table 1. These are the actual parameters of a rolling bearing, whose type is MB ER-10K. It will also be used as an experimental bearing in Section 5. The variable speed was set to $f_{r}=50+50t\;\;\mathrm{Hz}$, the natural frequency of vibration signal attenuation was $f_{n}=4000\;\mathrm{Hz}$, the sampling frequency was $f_{s}=20,000\;\mathrm{Hz}$, and the signal length was set to 1.5 s. The calculation yielded a fault characteristic coefficient of 3.0539 for the outer race.

**Figure 3:**
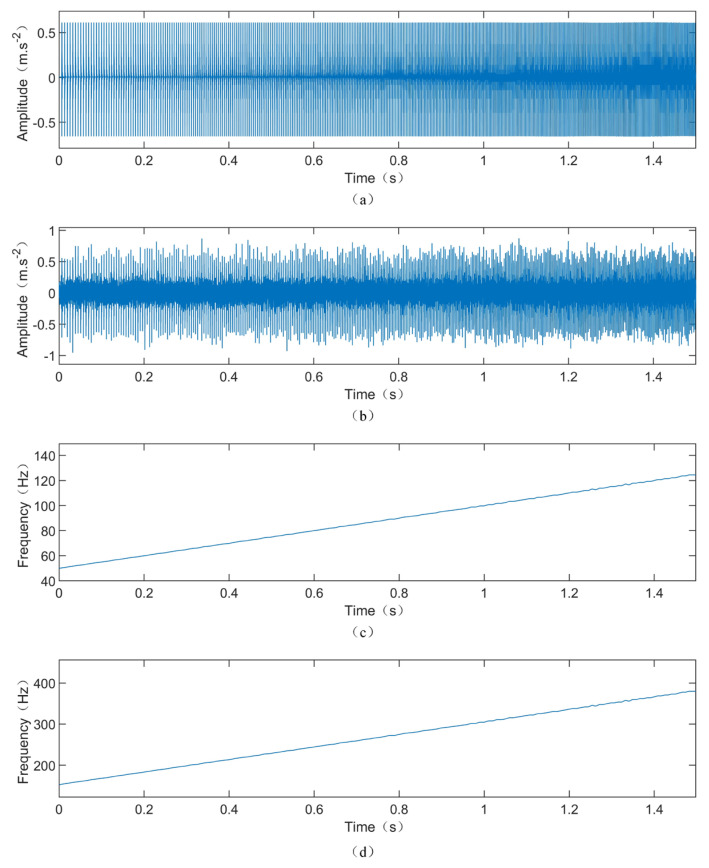
The simulated signal: (**a**) time-domain waveform of the simulated signal; (**b**) time-domain waveform of the simulated signal with noise; (**c**) instantaneous rotational frequency of the simulated signal; (**d**) fault frequency of the simulated signal.

The time-domain waveform of the vibration signal, instantaneous rotational frequency, and outer race fault frequency of the simulation model are shown in Figure 3a–d, respectively. It can be observed that the vibration signal exhibits repetitive impacts with consistent amplitudes. As the speed increases, the intervals between impacts gradually decrease. Figure 3c depicts the extracted instantaneous rotational frequency from the vibration signal, which linearly increases from 50 Hz, consistent with the model settings. Figure 3d shows the characteristic curve of the outer race fault frequency, increasing from 152.64 Hz to 379.98 Hz with the increase in speed. To make the simulated signal more closely resemble actual vibration signals, white noise with a signal-to-noise ratio of 10 dB was added. The time-domain waveform after adding noise is shown in Figure 3b.

**Figure 4:**
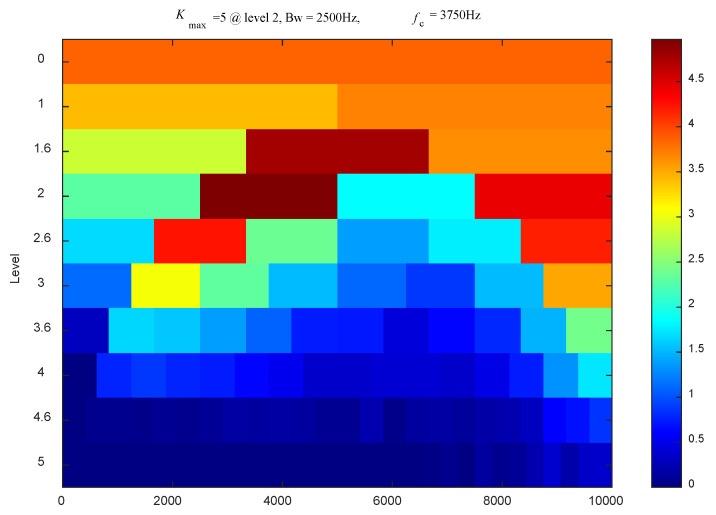
Kurtogram results of the simulated vibration signal with noise.

**Figure 5:**
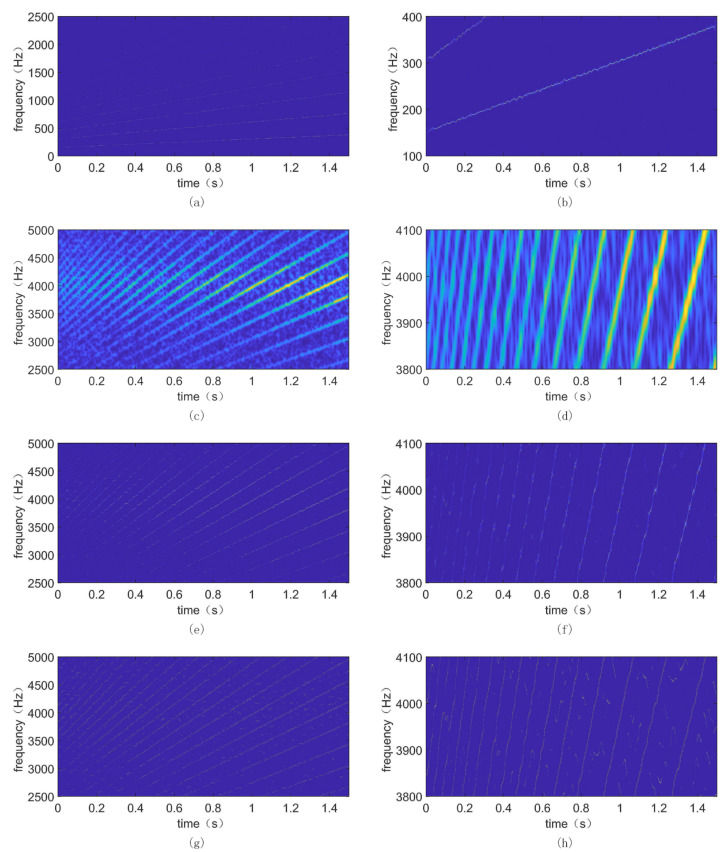
Time–frequency spectrum of different methods: (**a**) obtained using the proposed method; (**b**) partial enlarged of (**a**); (**c**) obtained using STFT; (**d**) partial enlarged of (**c**); (**e**) obtained using SST; (**f**) partial enlarged of (**e**); (**g**) obtained using MSST; (**h**) partial enlarged of (**g**).

The simulated vibration signal with noise was processed using the method of synchrosqueezed transform based on fast kurtogram and demodulation and PAA. First, the fast kurtogram method was employed to process the noisy vibration signal, and the result is shown in Figure 4. It can be observed that the optimal band has a center frequency of 3750 Hz and a bandwidth of 2500 Hz. Therefore, the optimal filtering band is from 2500 Hz to 5000 Hz. Then, the filtered signal was demodulated using the Hilbert transform to obtain the low-frequency envelope signal. Finally, under the same parameters (N = 1000, *σ* = 0.32), the SST method was applied to the filtered and demodulated signal, and the original signal was processed using the STFT, SST, and MSST methods, respectively. Their processing results and zoomed-in views are shown in Figure 5.

In order to quantitatively analyze the energy concentration of different methods, the Rényi entropy of the time–frequency results was calculated. The smaller the Rényi entropy value, the more concentrated the time–frequency representation. The results are shown in Table 2. The Rényi entropy for the proposed method is 4.0674, for STFT it is 12.4503, for SST it is 9.1264, and for MSST it is 5.5143. The Rényi entropy of the proposed method is significantly lower than that of the other methods, indicating that the time–frequency representation of the method is the most concentrated. Meanwhile, as seen from Figure 5, STFT exhibits energy divergence issues and fails to accurately identify fault frequencies. Although SST and MSST improve the time–frequency concentration compared to STFT, they are still affected by background noise, resulting in time–frequency blurring. Compared with the aforementioned methods, the method employed in this paper significantly reduces the influence of background noise, improves time–frequency concentration, and effectively displays the fault frequencies of variable-speed rolling bearings, consistent with theoretical expectations. Therefore, the method is effective and efficient for diagnosing faults in variable-speed rolling bearings.

In conclusion, the analysis of simulated signals validates the effectiveness of the proposed method and demonstrates its superiority compared to other methods.

Because the simulated signals have a relatively small amount of data, signal compression using PAA is not necessary within the computational limits of SST. Further analysis will be conducted on experimental signals in the next chapter.

## 5. Experimental Signal Validation

**Figure 6:**
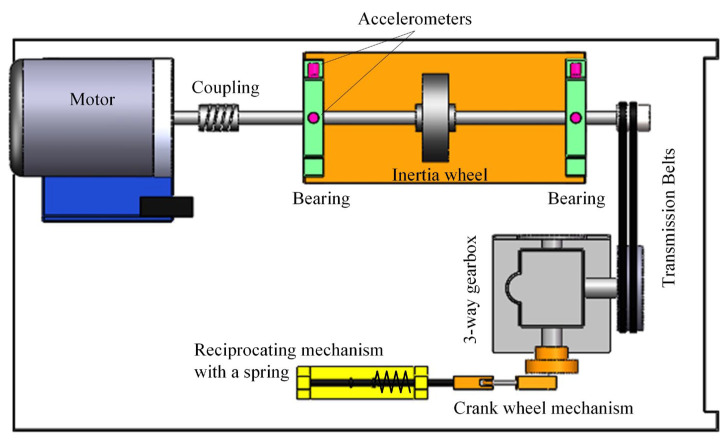
Schematic diagram of Mechanical Failure Simulation Bench.

**Figure 7:**
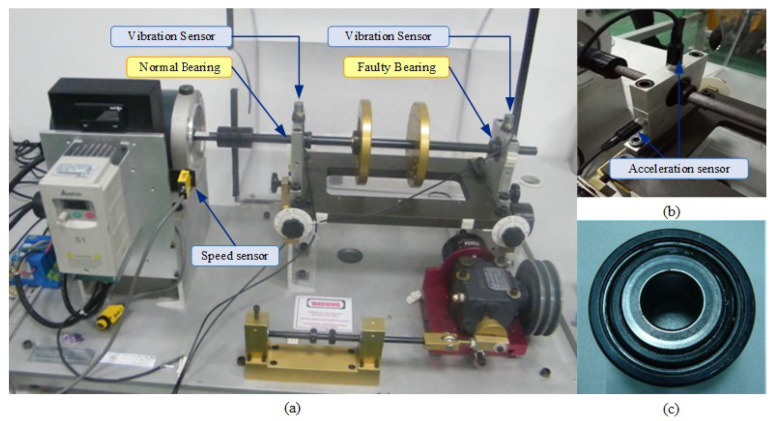
Mechanical failure simulation bench and faulty bearing. (**a**) Mechanical failure simulation bench. (**b**) Partial enlargement of (**a**). (**c**) Faulty bearing.

In this section, we validate the effectiveness of our proposed method using experimental signals. The experimental setup for simulating rolling bearing faults under variable-speed conditions is conducted on the test rig shown in Figure 6 and Figure 7. Figure 6 is a conceptual diagram, while Figure 7a,b contains photographs of the actual plant. The test rig consists of a motor, coupling, accelerometer, shaft system (shaft, bearings, turntable, etc.), transmission belts, three-way gearbox, crank wheel mechanism, control system, and data acquisition system. The speed of the motor can be adjusted within 10,000 rpm during the experiment. Three channels of signals are synchronously collected during the experiment, including two channels of vibration and one channel of speed pulse signals. Accelerometer sensors used for vibration measurement are installed on the bearing housing to measure radial vibrations in both the vertical and horizontal directions. The type of rolling bearing used is MB ER-10K, as shown in Figure 7c, with a 0.2 mm outer ring fault crack; the specific parameters are listed in Table 1.

**Figure 8:**
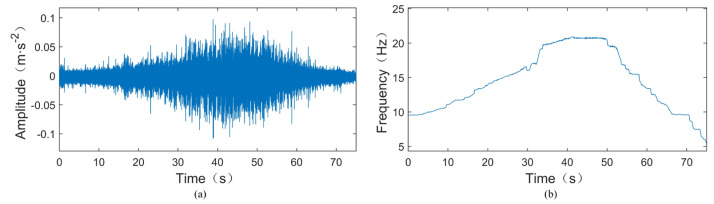
Time-domain waveform and instantaneous rotational frequency of the TEST-1. (**a**) Time-domain waveform of the TEST-1. (**b**) Instantaneous rotational frequency of the TEST-1.

During the experiment, the rotational speed was gradually increased from around 600 rpm to near 1200 rpm and then gradually decreased back to around 600 rpm. The sampling frequency was set to 25.6 kHz, and the sampling duration was 75 s. Figure 8a shows the original vibration signal of the test TEST-1, where a crack fault with a width of 0.2 mm is inserted into the outer ring of the tested bearing. According to Table 1, the outer ring fault characteristic coefficient of this bearing can be calculated as 3.052, indicating that the outer ring fault characteristic frequency *f*_BPFO_ = 3.052*f*_r_, where *f*_r_ is the rotational frequency. The instantaneous rotational frequency calculated from the rotational speed pulse signal is shown in Figure 8b. From Figure 8a, it can be observed that the amplitude of the vibration signal increases as the rotational speed increases. In such cases, time-domain statistical features such as RMS and kurtosis cannot be used to assess bearing faults.

**Figure 9:**
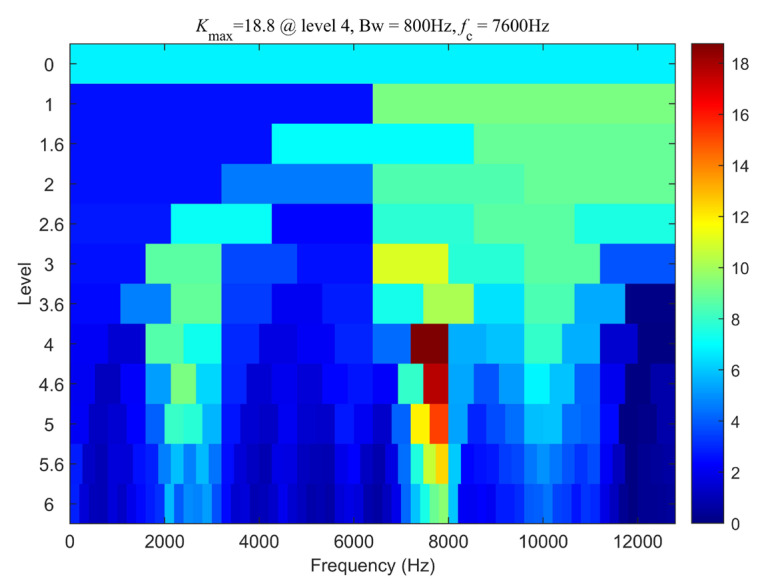
Fast Kurtogram plot of the TEST-1.

**Figure 10:**
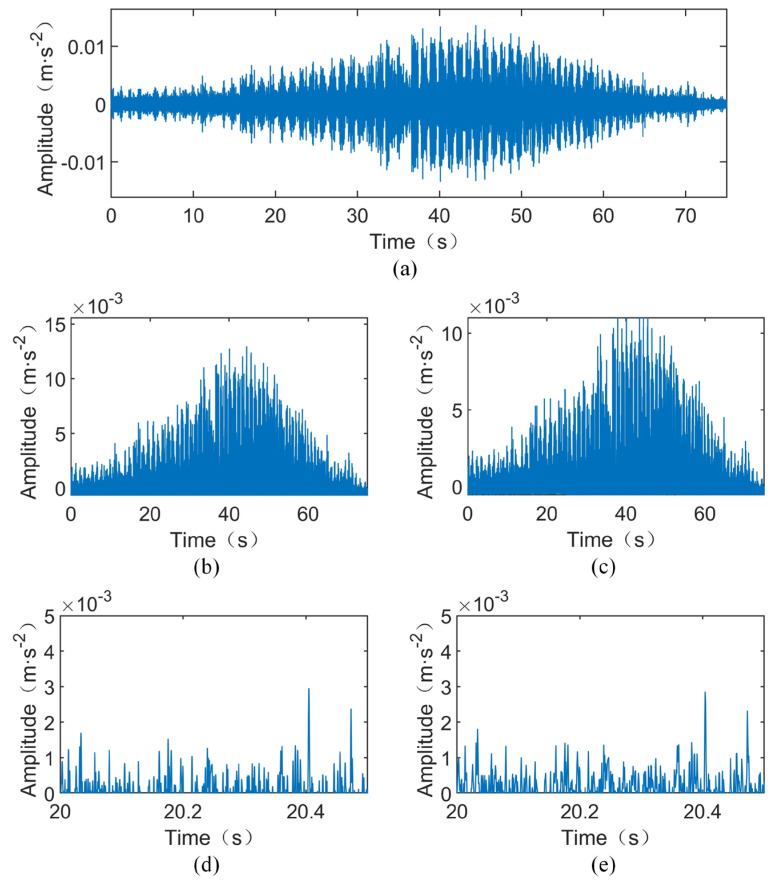
Time-domain spectrum of the filtered, demodulated, and compressed signal of the TEST-1. (**a**) Time-domain waveform after filtering of the TEST-1. (**b**) Time-domain waveform after filtering and demodulation of the TEST-1. (**c**) Time-domain waveform of the compressed signal of the TEST-1. (**d**) Localized enlargement of Figure (**b**). (**e**) Localized enlargement of Figure (**c**).

Analyzing the experimental signal using the bearing fault diagnosis method—synchrosqueezed transform based on fast kurtogram and demodulation and piecewise aggregate approximation—we first processed the vibration signal using the fast kurtogram algorithm. The result is shown in Figure 9. From the graph, it can be observed that the center frequency of the optimal demodulation band is 7600 Hz with a bandwidth of 800 Hz. Therefore, the optimal filtering band for envelope demodulation is chosen as 7200 Hz to 8000 Hz. The time-domain waveform after filtering is depicted in Figure 10a.

Next, the filtered signal is demodulated using the Hilbert transform to obtain the low-frequency envelope signal, as shown in Figure 10b. Subsequently, the PAA method is applied to compress the signal. As indicated in Figure 8b, the maximum rotational frequency of the shaft is 20.92 Hz, corresponding to a maximum fault frequency of 63.85 Hz for the outer ring fault. According to Equation (15), assuming a factor of three times the fault characteristic frequency components are retained in the PAA-compressed signal, i.e., *K* = 3, the PAA window length needs to satisfy w≤52.21. Considering the computational memory limits of the current computer while maintaining the integrity of the maximum preserved information, a window length of w=20 is chosen, corresponding to an equivalent sampling frequency of 1.28 kHz for the compressed signal, as depicted in Figure 10c. Zooming in on part of Figure 10b yields Figure 10d, while enlarging the compressed signal portion of Figure 10c produces Figure 10e. Comparing Figure 10b,c with Figure 10d,e, it can be observed that, although the amplitude of the compressed signal is smaller than that of the envelope signal, their waveforms are consistent.

**Figure 11:**
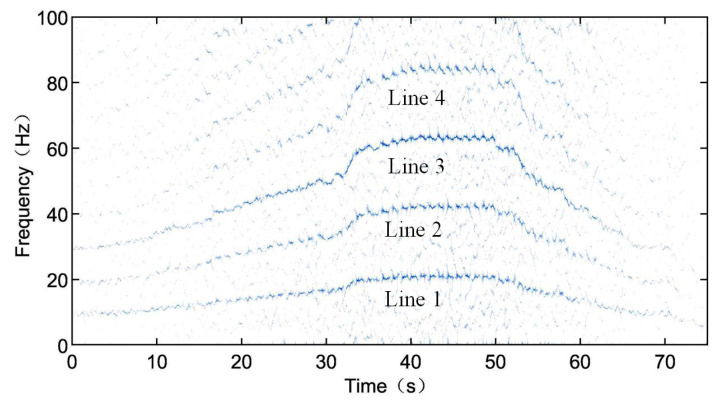
SST time–frequency spectrum of the enveloped and compressed signal of the TEST-1.

Finally, the compressed signal is subjected to SST processing, and the results are shown in Figure 11. In Figure 11, four clear frequency components can be observed, with an approximate harmonics relationship. The first, second, and fourth lines can be identified as the rotational frequency X (line 1) and its second harmonics 2X (line 2) and fourth harmonic 4X (line 4). Since the fault frequency 3.052X is close to three times the rotational frequency, line 3 may represent either the triple frequency 3X or the fault frequency 3.052X. Observing the amplitudes of the four lines, it can be noticed that from line 1, line 2, and line 4 that their amplitudes gradually decrease, but they are significantly smaller than line 3. Hence, it can be inferred that the third line not only represents the triple frequency of the rotational frequency 3X but also includes fault frequency components. Based on this, it is determined that the measured bearing is a faulty bearing.

**Figure 12:**
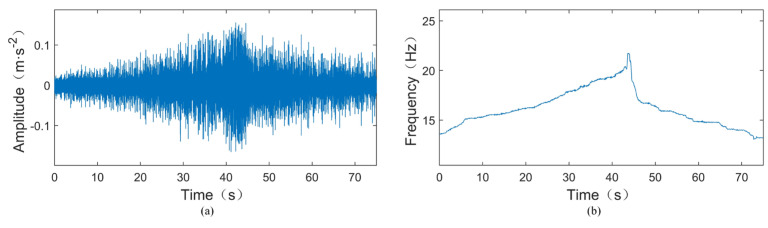
Time-domain waveform and instantaneous rotational frequency of the TEST-2. (**a**) Time-domain waveform of the TEST-2. (**b**) Instantaneous rotational frequency of the TEST-2.

**Figure 13:**
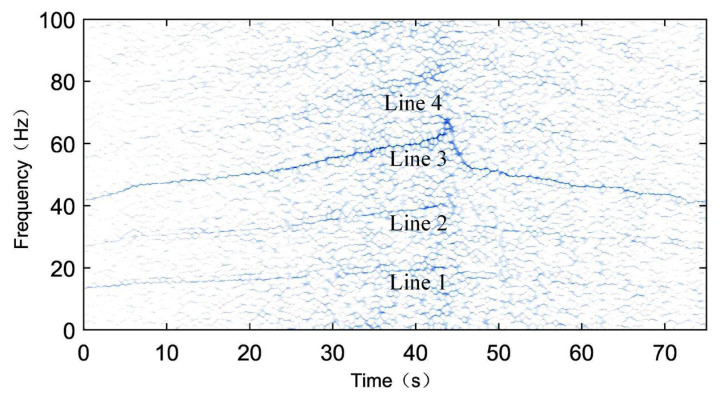
SST time–frequency spectrum of the enveloped and compressed signal of the TEST-2.

To further illustrate, we validate the proposed method using another experimental signal. Figure 12 shows the vibration signal and instantaneous rotational frequency of another signal, TEST-2. Similarly, using the bearing fault diagnosis method proposed in this paper—synchrosqueezed transform based on fast kurtogram and demodulation and piecewise aggregate approximation—the SST time–frequency spectrum of the compressed signal is shown in Figure 13. Observing Figure 13, it can be seen that there are only some vague noise points at the components of the rotational frequency X (line 1), its second harmonic 2X (line 2), and fourth harmonic 4X (line 4), while a clear spectral line is present at the triple frequency 3X (line 3). Since the amplitudes of the 1X, 2X, and 4X components are very small, the spectral line near the triple frequency 3X should represent the fault frequency component 3.052X, leading to the conclusion that the tested bearing is faulty.

**Figure 14:**
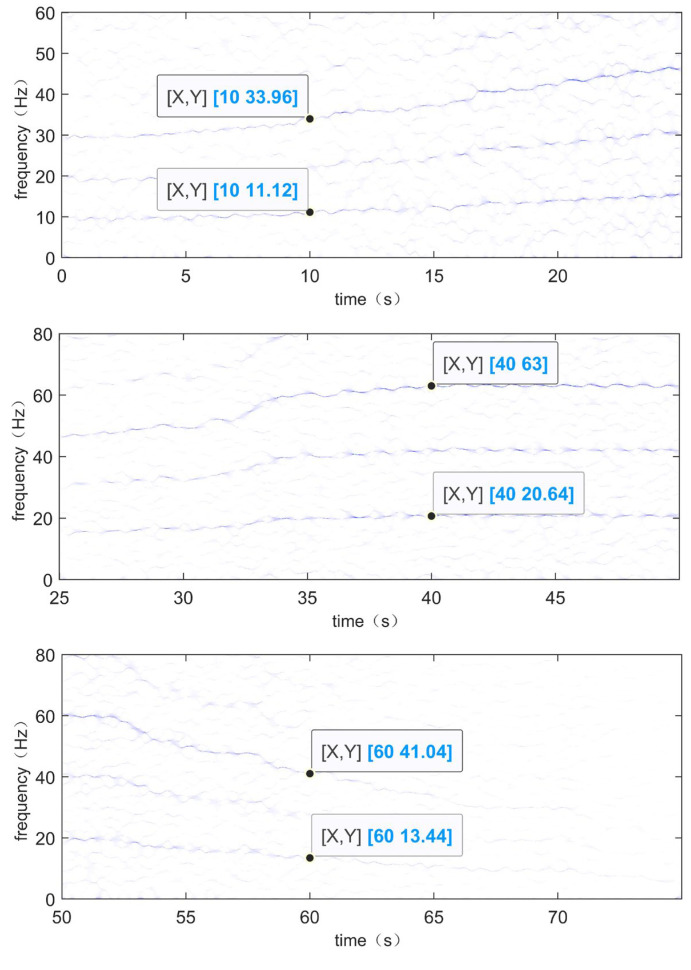
Localized enlargement of the time–frequency spectrum of TEST-1: period of speed increase (**top**), period of speed uniformity (**middle**), and period of speed decrease (**bottom**).

**Figure 15:**
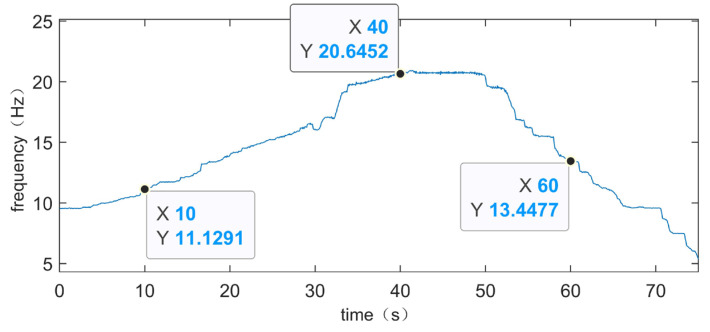
Vibration signal TEST-1’s instantaneous rotational frequency.

In order to confirm that the method can effectively identify fault frequencies, the time–frequency spectra of the vibration signal TEST-1 are locally magnified at three specific time periods, and the frequency values of the selected specific moments are compared with the theoretical values of the corresponding moments. The locally magnified time–frequency spectra of the vibration signal TEST-1 are shown in Figure 14, which corresponds to the period of increasing rotational speed, the period of constant rotational speed, and the period of decreasing rotational speed, respectively. The moments *t* = 10, *t* = 40, and *t* = 60 are selected, respectively, and it can be seen from the Figure 14 that when *t* = 10, the rotational frequency is 11.12 Hz and the suspected fault frequency is 33.96 Hz. At time *t* = 40, the rotational frequency is 20.64 Hz and the suspected fault frequency is 63 Hz. At time *t* = 60, the rotational frequency is 13.44 Hz and the suspected fault frequency is 41.04 Hz. The actual rotational frequency at the corresponding times can be obtained from Figure 8b, as shown in Figure 15. At *t* = 10, the actual rotational frequency is 11.1291 Hz; at *t* = 40, it is 20.6452 Hz; and at *t* = 60, it is 13.4477 Hz. As mentioned earlier, the fault feature coefficient of the experimental bearing is 3.052; so, theoretically, the corresponding fault frequencies at these times are 33.9660132 Hz, 63.0091504 Hz, and 41.0423804 Hz, respectively. The frequency resolution of this experiment is 0.04 Hz. The theoretical values and experimental results are within the error standard, demonstrating that this method can effectively identify fault frequencies.

In conclusion, the experimental results clearly show the trend of the fault frequency curve changing with time, which is generally consistent with the estimated fault frequency variations. This demonstrates that the proposed method has a good effect on handling the outer race fault frequencies of rolling bearings under the condition of variable rotational speed.

## 6. Conclusions

This paper proposes a variable-speed rolling bearing fault diagnosis method, synchrosqueezed transform based on fast kurtogram and demodulation and piecewise aggregate approximation. This method first performs bandpass filtering and demodulation on the vibration signal, then compresses the envelope signal using PAA, and finally applies the SST method to process the compressed signal to extract fault characteristic frequencies. The advantages of this method are its ability to reduce the influence of noise on the signal, improve time–frequency resolution, optimize the processing results of variable-speed rolling bearing signals, and overcome the limitation of the original method’s inability to handle long signals. Simulation model signals and experimental signal verification demonstrate that, compared to other SST methods, this method has the advantages of reducing noise, improving time–frequency concentration, and effectively diagnosing fault frequencies in variable-speed rolling bearings. In addition, the selection of parameters *K* and *f_max_* for PAA requires prior knowledge, bearing parameters, and rotational speed information. Further development is needed to address this issue.

## Figures and Tables

**Table 1:** Simulation bearing parameter table.

Z	d	D	D − d	D + d
8	7.9375 mm	33.5602 mm	25.6227 mm	41.4977 mm

**Table 2:** Rényi entropy results for each method.

Method	The Proposed Method	STFT	SST	MSST
Rényi entropy	4.0674	12.4503	9.1264	5.5143

## Data Availability

Data are contained within the article.
